# Scenarios to Improve the Patient Experience Time in a Tertiary Academic Hospital Using Simulation

**DOI:** 10.7759/cureus.30751

**Published:** 2022-10-27

**Authors:** Anas M Al Halabi, Elmukhtar Habas, Hafedh Ghazouani, Abdelsalam M Borham, Esmat Swallmeh, Abdul-Badi Abou-Samra

**Affiliations:** 1 Quality and Patient Safety, Hamad Medical Corporation, Doha, QAT; 2 Internal Medicine, Hamad General Hospital, Doha, QAT; 3 Qatar Metabolic Institute, Hamad Medical Hospital, Doha, QAT

**Keywords:** service quality, des, simulation, admission unit, pet duration, admission process

## Abstract

Background

Shortening the patient experience time (PET) in the emergency department (ED) improves patient quality and satisfaction and reduces mortality and morbidity. Worldwide, the PET target in the ED is ≤ 6 hours; however, the PET awaiting admission to inpatient Medicine at Hamad General Hospital (HGH) in the Qatar State, through ED is currently 15.3±6.4 (mean ± SD) hours.

Aim

Identify solutions to reduce the PET duration at HGH-ED to the international target.

Method

A cohort study was done using the Discrete-event simulation (DES) model, utilizing a commercial simulation software package (Process Model Inc., Utah, version 5.2.0). One-year data, January 1, 2019 - December 30, 2019, was analyzed and found to follow seven subprocesses. The duration of each subprocess was recorded, and the average time was calculated. A computer simulation scheme was developed for all the subprocesses of the actual PET duration. The simulated PET was validated, and scenarios were proposed and assessed for each subprocess separately and in combination,

A constructed simulatory design using an iterative process involving a construction model. This model starts with the logical organization of submitted tasks based on their cycle times. A subject-matter expert interview was conducted to determine the appropriateness and frequency of actions. The duration of each activity in the considered process was defined using a triangular distribution.

Results

The actual PET duration for the Medical Department was 15.3±6.4 (mean + SD) hours. The three most prolonged PET subprocess durations were in the referral to internal medicine, the decision to admit, and finding a free bed; these represent 17.9%, 53.8%, and 16.7% of the PET, respectively. Adding two physicians to each shift, which shortens the subprocess of the decision to admit, reduced the PET duration by 27.5%. Moreover, creating a new admitting team (unit) that takes care of new patients admitted to the ED reduced PET duration by another 12.5%. Combining these two scenarios reduced the average PET duration to only 10.2±0.5 hours. In addition to these scenarios, the PET can be further decreased to six hours by increasing the number of inpatient beds.

Conclusions

The simulated scenarios indicated that restructuring the medical teams, adding two physicians to each shift, and creating an admissions team dedicated to the ED would reduce the total PET duration to 10.2 hours, Furthermore, PET's further reduction to six hours is predictable by increasing the bed number.

## Introduction

Waiting times for elective care [1], and emergency interventions are considered major problems (such as overcrowding and long waiting time for medical care) in the healthcare system, and patient wellness, impaired care access, and patient dissatisfaction [2,3]. One reason for the delay in providing the best flow of health services is the administrative boundaries. The time elapsed between the patient presenting to the emergency department (ED) and reaching the allocated bed are system efficiency measure.

For over two centuries, policymakers have progressively tackled delay problems (such as bed availability, inside hospital transportation, nurse availability, and bed management system) before patients reach their assigned beds [4]. Supply and demand policies have been launched to address challenges caused by excessive waiting times [5]. Initiatives have been proposed based on urgency and management requirements for medium-or high-risk patients [4], or patient choice programs [6,7]. For instance, in 2001, Norway abstained from rules impeding provider referrals to a single district, hoping to create a competitive referral system for elective admissions and care [8].

Although there are varying crosstalk and optimism regarding the potential benefits of patient choice, multiple recent reviews have reported minor and insignificant evidence of the tremendous opportunities for such effects [6,9]. A UK single-hospital study investigating the impact of patient choice for the facility on patient waiting times noted that more options were significantly linked with shorter waiting times, and the quantitative effect was moderately improved [10]. However, the London Patient Choice Project data reported that waiting time was significantly reduced in all patients, including those enrolled in the project [7].

An assessment of the geographical transfer of patients between hospitals across administrative boundaries was illustrated in a Canadian study on patients waiting for elective surgery [11]. A 2020 study reported that implementing a public wait-time website reduced the number of patients who attended crowded hospitals and moved them to other less overcrowded hospitals, leading to a significant reduction in waiting time. However, it adversely affects waiting times in other hospitals [12].

Prolonged patient experience time (PET) causes significant ED congestion, consequent decrease in the quality of care, increased morbidity and mortality, a low threshold of patient satisfaction, extended hospitalization, increased bed occupancy, and increased pressure on ED bed availability [13,14,15]. Therefore, this study aimed to identify the subprocesses with the longest time in the current admission process and test the simulation changes to identify improvements that can be conducted at the lowest possible cost.

Simulation

Simulation has several types, including continuous, agent-based, and discrete events [16]. Over the past two decades, modeling and simulation have been used remarkably well in the global healthcare industry, which depends on finding and developing solutions to increase the efficiency of this vital sector. This sector's modeling and simulation start by addressing the flow of healthcare beneficiaries, emergency rooms, and wards to review and simulate diseases and epidemics for populations within a range. This platform is used to develop models for measuring, evaluating, analyzing, and predicting the performance of healthcare systems [17]. The data sources and calculations required to implement and operate the simulation model were used for implantation in real situations.

## Materials and methods

Current patient care and admission process

Patients admitted to the Hamad General Hospital (HGH) Emergency Department (ED) had multiple subprocesses. The sub-processes are registration and assessment by the ED nurse and ED doctor, who usually order preliminary investigations. A specialty physician was contacted to assess and decide whether to admit the patient after the assessment subprocess. However, the latter may contain additional queries. Step three involved coordinating bed management, setting the bed, and transferring the patient to their designated space. Any delay in these steps lengthens the patient's stay in the ED.

**Figure 1:**
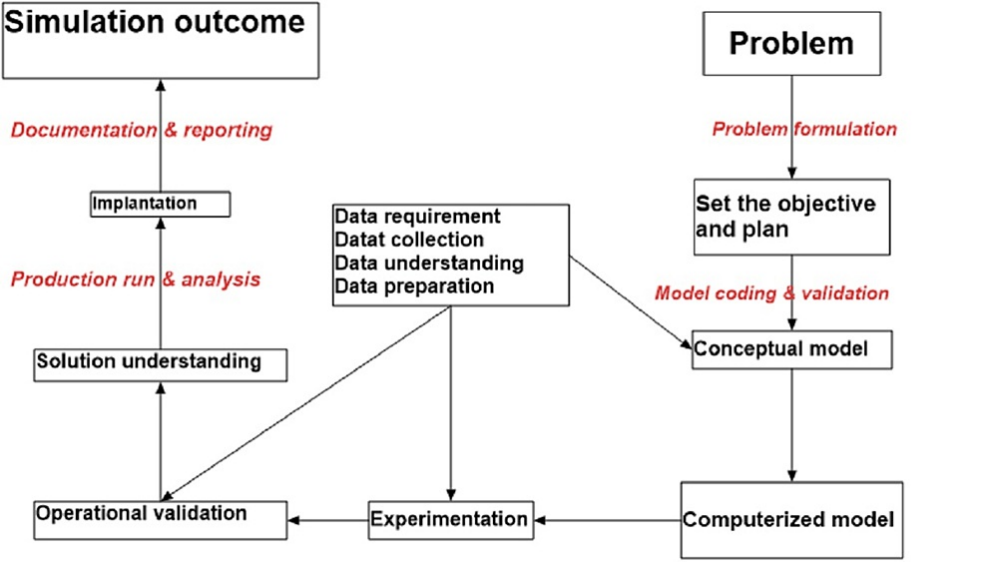
Proposed simulation model: Discrete-event simulation (DES) is the modeling of systems in which the state variable changes only at a discrete set of points in time

Discrete-event simulation (DES) is a system operation that separates a sequence of events in time. Every event at a selected point first marks a modification of the system state [12]. Among successive events, no change in the system is expected to appear; therefore, the simulation time will jump directly to the incidence time of the subsequent event, which is termed the next-event time progression. Thus, DES is a method for building models to simulate systems that depend on the distribution of imitation time as a function of future events at different times (Figure 1).

**Figure 2:**
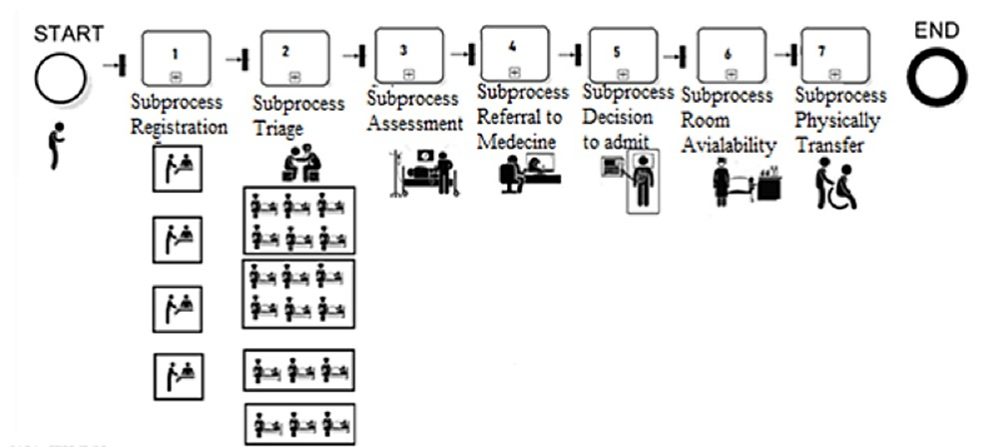
Context diagram of patient flow processes from ED to inpatient beds Inter-arrival time: Time rate (1), Percentage of time that the subprocess spends: Percentage (2), Registration (3), Triage (4), Assessment (5), Refer to Medicine Department in the emergency department (6), Decision to admit (7), Room availability and location (8), Physical transfer (9), Clerks unit (10), Nurses of the unit (11), Physician of the unit (12) duration.

This cohort project aims to use DES to explore the best scenarios that reduce PET and examine their impact on the different subprocesses of patient admission. DES is a technique used to represent real-world systems that may be divided into a series of conceptually distinct processes that independently advance through time. Each event in a particular process is given a logical time. This event's results may be passed on to one or more additional procedures. The outcome's content may result in the development of additional events to be handled at a future logical time. Therefore, due to the appropriateness of this system to the aim of the study, DES was used. Hence, we collected one-year data on the different components of the period spent in the ED for patients in whom a decision was made for admission to the medical department. Data were collected from the electronic record system (Cerner), physicians, nurses, bed managers, and administrative officers in the ED, between January 2019 and December 2019, and the relevant subprocesses were identified (Figure 2) and modeled. The electronic system (Cerner) that is used in the hospital is a closed system and the information can not be entered except by an official employee, and not possible to change the information saved. Furthermore, during the data collection, a significant number of interviews were conducted with patients, physicians, nurses, bed managers, other supportive teams (workers transferring patients), and administration employees about their thoughts to redirect the PET duration. The actual and model data were compared based on the number of physicians on the admission team and the number of patients waiting for transfer.

Statistical analysis

The patient transfer model from the ED to inpatient wards was implemented using a commercial simulation software package (Process Model Inc., Utah, version 5.2.0). Historical data were used to estimate the probability distributions of the model input data analysis using Easy Fit 5.6 Professional (Math Wave Technologies, 2015). Qualitative variables were described as frequencies and percentages.

Actual admission model & durations

PET, defined as the period from the patient's entrance to HGH ED reception until the patient is delivered to the assigned bed, includes seven subprocesses (Table 1 and Figure 2): registration, triage, assessment, referral to medicine, the decision to admit, allocation of bed, and physical transfer of the patient to the assigned bed. The human power capacity, ED bed capacity, and performance metrics are presented in Table 2.

**Table 1:** Model validation: Actual and simulated duration of PET subprocess duration P-value*:- After 180 days, the experimental simulation results were compared with the actual data of the system, and the comparison showed no statistical difference between the simulation results and the existing system. That t-test was significant at p<0.05.

Subprocess	Resource	Actual Data		Simulation	p-value*
		Average Duration (minutes, means ± SD)	Percentage of total PET	Duration deduced from the simulated model.	
Registration	Clerk	14± 1.9	1.4%	13.2	0.241
Triage	Nurse	17± 3.7	1.7%	16.7	0.849
Assessment	Physician	14.2± 4.5	1.4%	14.5	0.516
Referral to internal medicine	Medical registrar	175.3± 38.1	17.9%	177.2	0.641
Decision to Admit	Medical registrar	525.5±93.5	53.8%	526.8	0.773
Room available allocated	Nurse	163.6± 28.8	16.7%	165.4	0.427
Physically transfer	Nurse	66.4± 12.2	6.9%	66.3	0.513

**Table 2:** Parameters of actual operations with the base simulated model † Resource name (resource number of shifts and groups). †† A statistical distribution is a parameterized mathematical function that provides the probabilities of different outcomes for a random variable. Discrete and continuous distributions exist, depending on the arbitrary values of the models. * Calculated by measuring the percentage of time the subprocess spends and the amount of service time a process will need before it finishes. ^ Patient arrival rate Exp (5.78): The patient arrivals had a mean of 5.78 minutes.

Resource	Classification of available resources by type †	ED boarding^	Performance metrics ††
Registration	Four units 4 clerks by 4 units/shift		
	Four triage locations		
Triage	6 nurses in location 1/shift		
	6 nurses in location 2/shift		
	6 nurses in location 2/shift		
	6 nurses in location 4/shift		
Assessment	20-25 physician/shift		
Assessment	20-25 physician/shift		
Referral to internal medicine	1 medical physician/shift	Average daily admitted patients =50±14 patients	
Decision to Admit	1 medical physician/shift	ED bed capacity =250 beds	
Decision to Admit	1 medical physician/shift	ED bed capacity =250 beds	The median IQR overall PET* process was (17.0:9.4-20.5) hours, with 7.9 % of the admitted patients having a total admission duration was < 6 hours
Room available allocated	1 in-charge nurse/shift		
	1 nurse/shift		
	1-bed manager /shift		
	1 housekeeping/shift		
Physically transfer	2 in charge nurses /shift		
	1 nurse/shift		
	1 nursing aid		
Discharge	1 case manager/shift		
	1 physician /shift		
	1 nurse /shift		
	1 housekeeping/shift		

A process model is a computer model that mimics the dynamic behavior of a real process; it evolves to visualize and quantitatively analyze its performance. The process model is the most effective tool for performing quantitative 'what-if' analysis and plays different scenarios of the process behavior as its conditions and variables change with time. This simulation capability allows experiments to be performed on a computer display and test solutions to evaluate and determine their effectiveness before real implantation. Typical applications include staff and capacity planning, cycle time; throughput capability; and resource utilization.

Experimental (simulation) model

Formalized elements and mechanisms were identified as relevant by parameters and variables, using data observed over time. Historical data were created for patients' admission processes (Table 3 and Figure 2). A simulatory design was constructed using an iterative process involving model construction, which began with the logical organization of submitted tasks based on their cycle times, representing resource utilization. A subject-matter expert interview was conducted to determine the appropriateness and frequency of actions and to collect information on the professional rules restricting these actions. The interview involved the physician, nurses, bed managers, and administration officers. The duration of each activity in the considered process was defined using a triangular distribution, and the parameters used are listed in Table 3.

**Table 3:** The different tested scenarios

Scenario description	
Scenario 0: Base Model	Current situation (Without intervention)
Scenario1	Change to Base Model: Adding clerk in the registration subprocess
Scenario2	Change to Base Model: Adding nurses to the triage subprocess
Scenario3	Change to Base Model: Adding nurses to the assessment subprocess
Scenario4	Change to Base Model: Adding ED physician in referral to internal medicine subprocess
Scenario5	Change to Base Model: Adding only one physician in the decision to admit subprocess
Scenario6	Change to Base Model: Adding two physicians in the decision to admit subprocess
Scenario7	Change to Base Model: Adding nursing assistant in-room available subprocess
Scenario8	Change to Base Model: Adding nursing assistant in the physical transfer subprocess
Scenario9	Change to Base Model: Implementation of admitting team in ED

Different scenarios were proposed and tested to determine the best strategy for reducing patient admissions. Scenarios designed for a particular intervention added one or more changes to the subprocesses, and the influence of the intervention on the entire admission PET scan was assessed. The research team members proposed the scenarios based on the collected data and their personal views, experience in the facility, and knowledge about the common problems they faced. Furthermore, the simulation program used according to the given data suggests different practical solutions to reduce the PET duration. On these bases, nine different scenarios were proposed and simulated (Table 3). Scenarios were developed by adding one or more staff members to each subprocess to detect changes in the operational duration of the subprocesses and the duration of the entire process. After testing each scenario's effect on the PET duration, the combination of two or three scenarios that affected the PET significantly was tested to detect the best combination that improved the PET duration. The developed simulation model was validated, verified to be representative, and used to determine the best scenario for reducing PET in patients. Accordingly, visual tracking of each group of blocks was verified to ensure that the care path was correct. Furthermore, senior managers validated the conceptual and generic simulation models.

A simulation model was created for approximately 3,700 simulated patients over the course of three months. Accordingly, the simulation model was driven by real-time and actual patient data. The major sources of data were electronic records (Cerner), firsthand observations, and staff interviews (nurses, physicians, bed managers, and administration officers). The simulation model was routinely calibrated and executed for 180 days. To decrease system variation caused by chance, ten replications were carried out. The tested model accurately represented the system, resulting in a PET duration for admission that was comparable to the observed data. The simulation is as realistic as feasible due to the average operational parameter values recorded throughout the experiment. The simulation model was constructed using operational parameters (Table 2).

## Results

**Figure 3:**
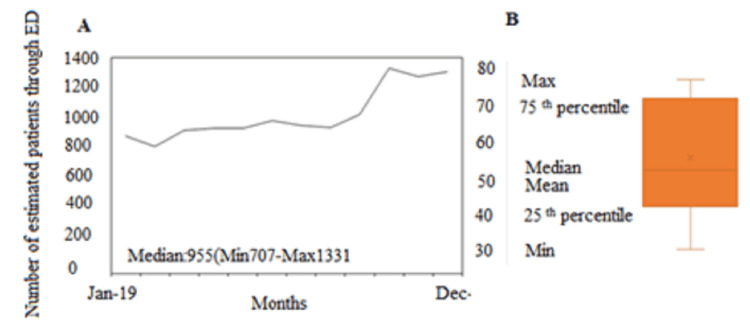
The number of patients admitted to internal medicine. A. Monthly admission and B. Daily admission (average, median, and 25th – 75th percentile ranges are shown)

In 2019, 386,889 patients visited the ED, where 30,185 patients were referred to the medical teams for admission to internal medicine. The decision to admit 19,058 patients was made, corresponding to an average of 52±28 admissions per day (range 28-79 patients). The monthly admission ranged between 707-1331 patients (median of 955 per month) (Figure 3).

The observed PET duration was 15.36.4 (mean ± SD) hours, corresponding to a median of 17 hours and an IQR of 9.4-20.5 hours. The most extended PET subprocess durations were noted in the referral to internal medicine and the decision to admit and find a free bed, representing 17.9%, 53.8%, and 16.7% of PET, respectively (Table 1).

**Figure 4:**
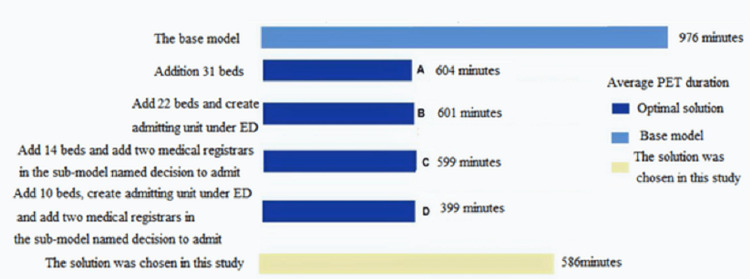
Logical solutions and Actions that ensure the optimization of minimizing the PET to less than six-hour and achieving 100% performance

The impact of changing the numbers of doctors, nurses, and beds was examined in nine scenarios (Table 3). Each scenario resulted in PET improvement; however, combining scenarios six and nine (new scenario 10 in Table 4) further reduced the PET with minimal extra cost (Table 4 and Figure 4). Scenarios six and nine were chosen because scenario six improved the PET by 27.5% and scenario nine reduced the PET by 12.5%. Therefore, their combination (scenario nine) improved the PET by 40%. The minimal cost increase is possible only if two new physicians are hired, but if we used the already working physicians, there will be no significant extra cost.

**Table 4:** Results of scenarios compared to the base model (in minutes) Patient experiment time (PET), Minutes (mins)

Designed scenario (Change to Base Model)	Average Total PET	Improvement in minutes (% of decrease)	Rank
Scenario0	976 mins	Without intervention	Base Model
Scenario1	970 mins	6, (0.5%)	10
Scenario2	965 mins	11, (1.0%)	8
Scenario3	967 mins	9, (0.8%)	9
Scenario4	907 mins	69, (7.0%)	5
Scenario5	781 mins	195, (19.9%)	3
Scenario6	712 mins	264 (27.5%)	2
Scenario7	947 mins	29 (2.87%)	6
Scenario8	952 mins	22, (2.36%)	7
Scenario9	849 mins	28, (12.5%)	4
Combined scenario 10 ( it includes scenarios 6 and 9)	586 mins	390, (40%)	1

Furthermore, adding the number of beds will improve the PETas illustrated in Figure 4.

## Discussion

**Figure 5:**
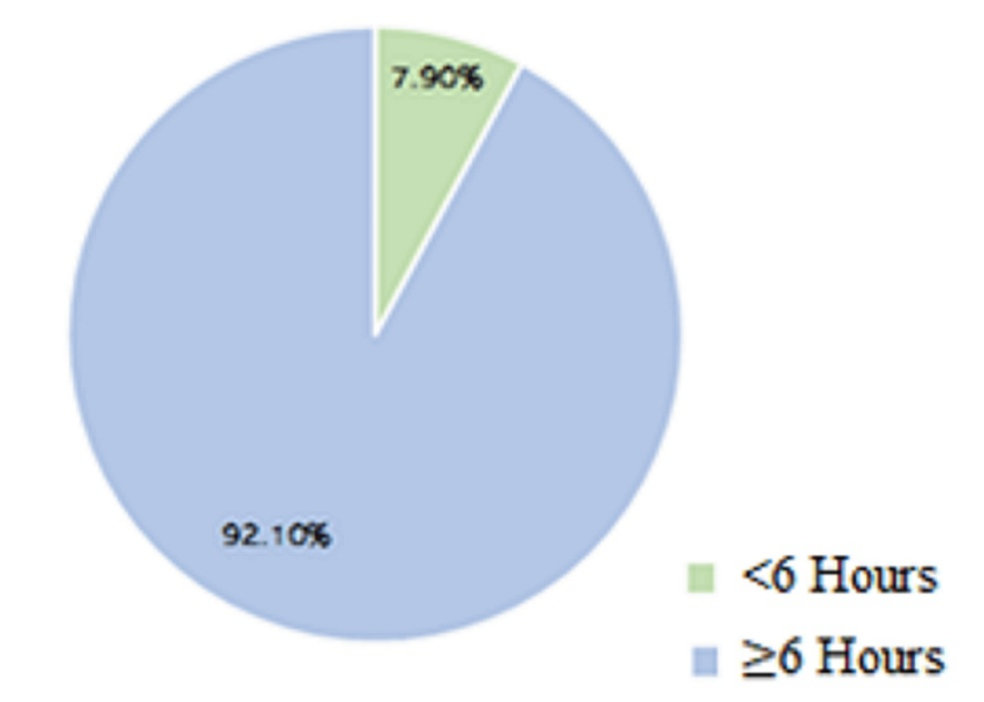
Distribution of the admitted patients in medicine wards via the emergency department per PET duration within the six-hour target

The total number of 248 inpatient beds allocated to medical patients included 51 beds (20%) for the acute medical assessment unit (AMAU), 24 beds (10%) for the acute assessment unit (AAU), and 173 beds (70 %) as inpatient wards. The AMAU is used to admit a high-turnover patient with a predicted length of stay of fewer than 72 hours and is located within the main hospital tower. AAU is used to rapidly assess patients likely to be admitted for a shorter period for observation, quick investigations, or planned procedures. The admission percentage distributions among the three internal medicine sections were 45%, 35 %, and 20% of the main internal medicine ward beds, AAU beds, and AMAU beds. The average duration of the admission process shows a PET of 17 hours (Table 1). The time study data analysis showed a normal parametric distribution (used to model the service times). Only 7.9% of the admitted patients had a total admission duration within the six-hour target (Figure 5).

The simulated operational changes that positively impacted all measured metrics are listed in Table 4. The admission process was improved by adding two medical registrars to the decision to admit the stage by 27.5% (scenario 6). Adding a medicine-admitting team to the ED reduced the total admission time by 12.5%. Combining the two interventions (scenarios six and nine) reduced the PET by 40% (i.e., from 17 to approximately 9.8 hours). The functions of the medical admitting team within the ED are to make an admission decision, care for admitted patients and patients awaiting admission, and move the patient to the final destination immediately or within 48-72 hours, thereby preventing ED congestion and reducing PET. Implementing the admitting team model brings high-level collaboration between the ED and other HGH services, resulting in a closer and more efficient work relationship, allowing more efficient collaboration between these service teams and improving PET. The admitting team creates a functional bond through physical proximity, where members of the admitting team can interact and efficiently exchange operational options and knowledge. The admitting team can be created by redistributing and reallocating the physicians. However, the impact of this redistribution on other clinical areas is unknown and may need further analysis.

Optimization and simulation based on a constructed mathematical model were further utilized to improve PET to the recommended target (six hours). Four scenarios were suggested: adding 31 beds to the inpatient (scenario a); adding 22 beds, creating an admitting team (scenario b); adding 14 new inpatient beds and two medical physicians in the admitting decision subprocess (scenario c), and adding only ten new inpatient beds, plus an admission team and two medical physicians in the decision to admit subprocess (scenario c). Testing these four scenarios (a, b, c, and d) revealed that none improved the PET to the target (six-hour) (Figure 5). Therefore, many possible changes can be made to improve PET.

## Conclusions

Adding two physicians to each shift in the decision subprocess to admit and install the admitting team is worth testing because it improves PET duration without adding a significant additional expense if we used the available working forces, but certainly, if more physicians are needed, an extra cost might be needed. However, expanding inpatient bed capacity increases bed availability and significantly reduces PET and ED overcrowding may require additional funding. As a result, either of these strategies to improve PET in Hamad General Hospital is recommended to improve service quality, morbidity, mortality, and patient satisfaction. Although the results seem promising, it remains a simulation (not a reality), which is a study limitation. Further real testing of these scenarios will be informative and may explore other issues, such as the need to hire new physicians for the admitting team and the ED. 
